# The World Health Organization Recommendations for Trachoma Surveillance, Experience in Nepal and Added Benefit of Testing for Antibodies to *Chlamydia trachomatis* pgp3 Protein: NESTS Study

**DOI:** 10.1371/journal.pntd.0005003

**Published:** 2016-09-21

**Authors:** Andrea I. Zambrano, Shekhar Sharma, Kathryn Crowley, Laura Dize, Beatriz E. Muñoz, Sailesh K. Mishra, Lisa A. Rotondo, Charlotte A. Gaydos, Sheila K. West

**Affiliations:** 1 Wilmer Eye Institute, Johns Hopkins School of Medicine, Baltimore, Maryland; 2 National Trachoma Program, Nepal Netra Jyoti Sangh (NNJS), Kathmandu, Nepal; 3 RTI International, Washington, DC, United States of America; 4 International Chlamydia Laboratory, Johns Hopkins Hospital, Baltimore, Maryland, United States of America; University of California San Francisco, UNITED STATES

## Abstract

**Background:**

The World Health Organization (WHO) now requires a second surveillance survey for trachoma after an impact assessment has found follicular trachoma (TF) <5% to determine if re-emergence has occurred. *Using* new WHO guidelines, we undertook surveillance surveys, and determined the prevalence of infection and antibody positivity, in two districts in Nepal.

**Methods:**

20 clusters were randomly selected within each district, 15 were randomly selected for antibody testing. In each cluster, we randomly selected 50 children ages 1–9 years and 100 adults ≥15 years. TF and trachomatous trichiasis (TT) were evaluated. Conjunctival swabs to test for chlamydial infection using GenXpert platform were obtained, and dried blood spots were collected to test for antibodies to *Chlamydia Trachomatis* pgp3 using the Luminex platform.

**Findings:**

3 cases of TF were found in the two districts, and one case of infection. Pgp3 antibody positivity was 2·4% (95% confidence interval: 1·4%, 3·7%), and did not increase with age (*P* = 0.24). No clustering of antibody positivity within communities was found. TT prevalence was <1/1,000 population.

**Interpretation:**

The surveillance surveys, as proposed by WHO, showed no evidence for re-emergence of trachoma in two districts of Nepal. The low level and no significant increase by age in seroprevalence of antibodies to *C trachomatis* pgp3 antigen deserve further investigation as a marker of interruption of transmission.

## Author Summary

Once districts have shown that the prevalence of follicular trachoma (TF) in children ages 1–9 years is below 5%, they must monitor for re-emergence. The World Health Organization (WHO) recommends a second surveillance or “pre-validation” survey to determine if re-emergence has occurred. Using new WHO guidelines, we undertook surveillance surveys, and determined the prevalence of antibody positivity, in two districts in Nepal. 20 clusters were randomly selected within each district, 15 were randomly selected for antibody testing. In each cluster, 50 children ages 1–9 years and 100 adults ≥15 years were randomly selected. Follicular trachoma (TF) was evaluated, conjunctival swabs to test for chlamydial infection were obtained, and dried blood spots were collected to test for antibodies to pgp3 in children. Adults were evaluated for trachomatous trichiasis (TT). Only 3 cases of TF were found in the two districts, and one case of infection. Overall Pgp3 antibody positivity was low (2·4%), it increase with age, and no clustering within communities was found. TT prevalence was <1/1,000 population. Our surveillance surveys, as proposed by WHO, showed no evidence for re-emergence of trachoma in two districts of Nepal. The low level and not significant increase by age in children with positive antibodies to *Chlamydia trachomatis* deserve further investigation as a marker of interruption of transmission.

## Introduction

Trachoma, a chronic conjunctivitis caused by repeated episodes of *Chlamydia trachomatis*, is still the leading infectious cause of blindness worldwide[1]. The World Health Organization (WHO) recommends a multi-faceted strategy, the SAFE strategy, for trachoma control (SAFE is Surgery for trichiasis, Antibiotics to reduce infection, Facial hygiene, and Environmental change for sustainable interruption of transmission)[1–3]. Country programs are encouraged to map suspected trachoma endemic districts to determine the prevalence of follicular trachoma (TF) and trichiasis (TT), and institute SAFE where trachoma is more than 5% in children ages 1–9 years and TT is 1/1,000 total population or more[1–4].

The goals are sustainable reduction in prevalence of TF in children ages 1–9 years of <5%, and the reduction of TT unknown to the health system of <1/1,000 population. After 3–5 years of SAFE implementation, impact assessments are carried out to determine progress, and if the impact assessments show achievement of goals, then vertical program activities, particularly around mass treatment with antibiotics, can cease. However, to ensure longer term, sustainable, achievement of goals, post-MDA surveillance in formerly endemic districts should be carried out. The approach to surveillance activities has varied widely by country, for example there is a National Trachoma Surveillance unit in Australia that reports annually from aboriginal at-risk communities[5] while in Oman, active trachoma was integrated into the surveillance of other communicable disease and special trichiasis screening was done in formerly endemic regions[6,7]. In September 2014, the WHO recommended the surveillance survey consist of a population-based prevalence survey at least 2 years post MDA, measuring again TF and TT[8]. There is only one publication describing the results using the new WHO guidelines, from a district in Tanzania where four years after cessation of MDA, trachoma did not re-emerge[9].

There is concern that when TF declines to such low levels there is the risk of overcalling by graders (due to the challenge of standardized training), or that follicular disease may be due to other causes, resulting in ongoing or re-starting interventions that are inappropriate and unnecessary and may waste resources that could be allocated to other areas. Additional data provided by a test for presence of infection, or a test for antibody positivity to *C*. *trachomatis* antigens, could be important additions to these surveillance surveys. However, there are insufficient data on infection and a test for antibody positivity to determine their usefulness in that scenario. A district wide surveillance survey in Tanzania found prevalence of TF of 0·4% was associated with a similarly low rate of infection, 1%, and overall rate of antibody to pgp3 positivity of 7·5%[6]. In that study, the TF prevalence in 1–3 year children, born after the trachoma program in that district had ceased, was 5%, and in more than half the communities, no child of in that age group had antibodies to pgp3. A significant increase by age in seroprevalence was observed, but not as pronounced as in other, currently endemic settings[10,11]. These data were suggestive of an important role for a test for chlamydial antibodies, which could be a marker of cumulative exposure to trachoma, but more data are clearly needed.

Nepal has reached its program goals in all of its formerly endemic districts, and is embarking now on surveillance surveys using the new WHO guidelines. We had the unique opportunity to add into these surveys a test for infection and a test for antibody positivity to *C*. *trachomatis* antigen pgp3. The goal of this study was twofold: first, using the new WHO surveillance recommendations, to determine the prevalence of TF and TT in two districts in Nepal which had ceased mass drug distribution for trachoma 2 to 4 years previously (continuing trichiasis case finding and treatment). Secondly, to determine the prevalence of chlamydial infection, and antibody positivity. This is the first country to fully implement and report data from the new WHO surveillance guidelines, which will enable better understanding of the timeframe for surveillance and the relationship between TF and potential ancillary tests for potential use in the future.

## Materials and Methods

### Ethics statement

This study was approved by the Johns Hopkins Institutional Review Board, the Nepal Netra Jyoti Sangh and the Nepal Health Research Council (238/2014). It followed the tenets of the Declaration of Helsinki. Written informed consent was obtained from the guardians of each child in the research project. All adults provided written consent for their examination and participation of themselves and their children participating in our surveys.

### Setting

Nepal is divided into 75 districts of which 20 are formerly endemic for trachoma and four are considered “high risk districts” because of residual TF (but <5%) after the last impact survey. The 20 districts contain villages called Village Development Committees (VDC) which is an administrative unit that can vary from 300 persons to 35,000. The VDC are further divided into wards, of which there are at least 9 per VDC.

Two districts were chosen for this study, Dang and Dailekh, where residual TF at 1·1% was found at the last impact survey conducted 2 and 4 years ago and surveillance surveys were planned for early 2015. They each had four rounds of Mass Drug Administration before program activities ceased.

### Random selection of clusters

Within each district, 20 clusters (see below) for the surveillance survey were randomly selected with probability proportional to size. Fifteen were randomly assigned to have a test of infection and test for antibodies. The remaining five had the clinical survey alone.

The steps for the selection of the clusters are described below:

1. A complete list of wards within chosen districts and the corresponding population was provided by the national census conducted in 2011[12].
2. The total population of the wards was reviewed: a) wards with a population above 300 were divided geographically to create several clusters in such a way that each cluster contained between 150 and 300 people; b) wards with population between 150 and 300 were kept as is c) wards with population below 150 were combined to get to the target population.
3. Clusters were defined as the geographical area with a target population between 150 and 300. (A geographical area with total population between 150 and 300 is expected to yield at least 50 children between the ages of 1 and 9 years).
4. Twenty clusters were randomly selected from each district for the surveillance survey, and the first 15 of these 20 randomly selected were assigned to the study of infection and antibodies.

### Study population

All residents of the geographic cluster were invited to the survey. Community volunteer went house to house after a random start and registered up to 50 children ages 1–9 years and 100 adults ≥15 years old for the survey.

Fifty children ages 1–9 year each from the 15 research VDC/district were included in our study of infection (1500 children) and within the 1500, only children ages 1–4 and 9 years were included in the study on antibodies. Data from 100 adults in each of the 20 VDCs/ district were included (4000 adults).

### Survey

The survey involved three components: bilateral grading for TF, conjunctival swab of the right eye for testing of *C*. *trachomatis*, and a finger prick for collection of blood spot for antibody testing. NNJS provided azithromycin treatment for TF cases.

A GTMP certified grader conducted the examination for TF, using the WHO simplified grading scheme[13], a torch and 2·5 loupe. All cases were re-affirmed by a second grader (AZ). The right upper eye lid was everted and a dry swab taken of the upper conjunctiva. Strict adherence to protocol was observed to avoid field contamination, and 3 control swabs per cluster were taken in the field to monitor possible contamination. These were labeled and analyzed in an identical fashion to true specimens. The swabs were placed in a Cepheid transport media tube (Cepheid, San Jose, CA), kept cold in the field and processed the next day by a trained technician in Nepal using the Cepheid GeneXpert platform. Results were reported as positive or negative. Known positive and negative controls (Zeptometrix Corporation, Buffalo, NY) were run weekly to check the machine. Blood was collected by finger prick from each child onto filter papers with six circular extensions, each calibrated to collect 10 μl of whole blood (TropBio Pty Ltd, Townsville, Queensland, Australia). These were dried and shipped to the Johns Hopkins University. The blood spots were analyzed for antibody to chlamydial antigen pgp3 as previously reported[10], using a multiplex bead assay on a Luminex 100 platform. The results are reported as median fluorescence intensity minus background (MFI-BG, where background is the signal from beads with buffer only) and the positivity cut-off was as determined by receiver operator characteristics (ROC) analyses[7]^.^

### Data analyses

The overall district prevalence of antibody was estimated as the mean of the individual cluster prevalence. Since there were so few cases of TF and infection, we just report the number of cases. The rate and the 95% confidence intervals using exact estimates for a binomial proportion are presented for antibody positivity and presence of TT. The proportion of children positive for antibodies against pgp3 is presented for each age category, and then for the 9 year olds. We compute confidence intervals around the proportion assuming a poison distribution. A test for trend (Mantel-Haenszel Chi-Square (1df)) was used to test for increase antibody positivity with age. The magnitude of the clustering of antibody positivity within sampled cluster was assessed using the intra-class correlation coefficient, the point estimate and 95% confidence interval is reported.

In adults, bilateral examination of TT was conducted; if identified, we everted eyelids for evaluation of scarring to relate the trichiasis to evidence of trachoma, and exclude other causes of trichiasis. Contact with healthcare system was determined by asking if they had had surgery before, or had been offered surgery before.

Role of Funding Source: Funding source/s had no role in the design or implementation of this study.

## Results

### TF and antibodies in children

A total of 2021 children were enrolled from the two districts in the surveillance survey. The prevalence of TF was 0·1% in Dang (95% CI = .03%, 0.55%) and 0·2% in Dailekh (95% CI = .02%, 0.72%). 1511 children from both districts were enrolled from the 15 clusters in the study, of whom 52% were male. One case of infection was detected in Dailekh district. No control swabs were positive.

**Fig 1:**
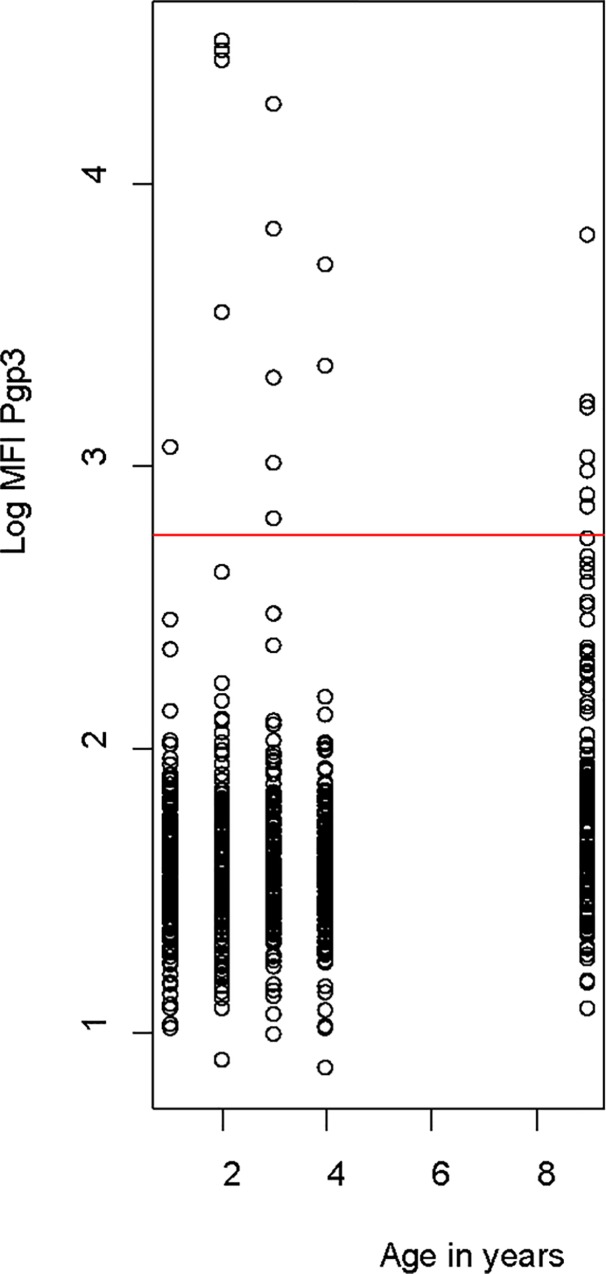
Log MFI–BG value for antibody to pgp3, distributed by age. The red line corresponds to the cut-off value for positivity. Of the 30 clusters studied, 17 (57%) had no antibody positivity, 8 (27%) had just one child positive, and 5 (16%) had two to three children antibody positive. No cluster had more than 3 children who were antibody positive (Fig 2). There was no evidence that children who were antibody positive clustered within community (ICC 95% Confidence interval: 0·05 (-0·21, 0·32), *P* = 0·68).

Within the 15 clusters, we tested 794 children for antibodies to pgp3 (Table 1). The prevalence of antibody positivity was low in both districts, 2% in 1–4 year olds. Among the nine year olds, the prevalence of antibody positivity was higher, 4% and 3% respectively, but the differences were not statistically significant (Fischer’s exact test *P* >0·05). There was no evidence of an increase by age in seroprevalence among the 1–4 year olds either (Table 1). Fig 1 shows the values by age of antibody positivity.

**Fig 2:**
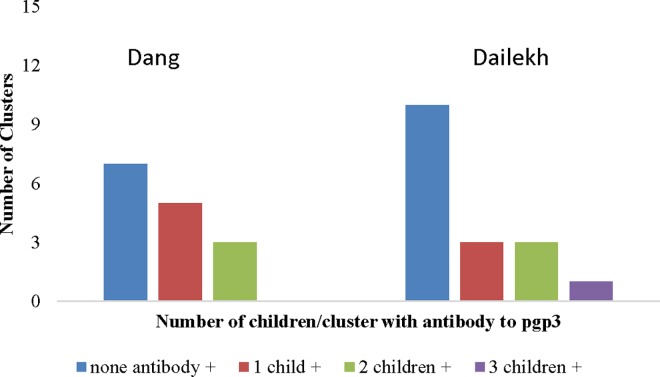
Number of clusters per district with zero, one, or more than one child antibody positive.

**Table 1:** Percent of antibody positivity stratified by age and district.

DANG			DAILEKH		OVERALL*	
Age in years	N	% pgp3 Ab positive	N	% pgp3 Ab positive	N	% pgp3 Ab positive
**1**	94	1·1	51	0	145	0·7
**2**	81	2·5	56	3·6	137	2·9
**3**	90	4·4	68	1·5	158	3·2
**4**	79	0	70	2·9	149	1·3
**1–4**	**344**	**2·0%**	**245**	**2·0%**	**589**	**2·0%**
**9**	**94**	**4·3%**	**111**	**2·7%**	**205**	**3·4%**

* Test for trend among 1–4 year olds, P = 0.24

### TT in adults

A total of 4,000 adults were examined in the surveillance survey, 2000 per district. 11 cases of trichiasis were found, of whom 4 were non trachomatous trichiasis (no evidence of scarring). Of the remaining 7 cases, 5 had been approached for surgery before and had refused (and refused again) so were already known to the health system. Two were new cases in Dang, who were referred for surgery (Table 2). The prevalence of TT in the total population was zero in Dailekh and 0·5/1,000 in Dang, indicating having achieved the goals for TT during the surveillance period.

**Table 2:** Trichiasis and TT in adults ≥15 years old stratified by district.

District	N total trichiasis cases	Trichiasis NOT due to trachoma	TT already known to the system	TT new cases	Rate of TT per Total population (exact Confidence Interval)
**DANG**	10	4	4	2	0·5/1,000 (0·1/1000-1·2/1000)*
**DAILEKH**	1	0	1	0	0%

* Exact Binomial CI

## Discussion

In this study from a country implementing the new WHO recommendations for post MDA surveillance in formerly endemic districts, we found no evidence of re-emergence of trachoma or infection in districts two and four years from MDA program cessation. Moreover, there was no evidence of exceeding the guidelines of one or more cases of TT unknown to the health system/1,000 population, in a setting with ongoing case finding and surgery that is part of the Nepal health care system.

Ideally, surveillance for an infectious disease should demonstrate ongoing interruption of transmission. However, we have no transmission markers for trachoma, and no agreed upon definition of interruption of transmission. The level of infection below which transmission cannot be sustained is unknown. In this study, we found only one case of infection in 1500 samples, whereas in a Tanzania setting where, after four years post MDA, clinical trachoma rates were still less than 1% TF, infection rates were 1·1%[9]. In neither site had trachoma specific activities in Mass Drug Administration, or environmental change been implemented in the four years (two years for Dailekh) preceding the surveillance surveys. A promising marker is antibodies to Chlamydial antigens, as they may show cumulative exposure to infection, and, if were very low or zero in children born during the surveillance period, could indicate interruption of transmission[9,10]. In Tanzanian communities with ongoing infection transmission, there was a sharp increase in the age seroprevalences of antibody to pgp3[10], such that by age 6 years, 100% of children had antibodies. In the current study, we observed a much lower prevalence of antibody positivity, 2%, and a not significant age specific increase. The low age specific distribution may reflect an absence of exposure and/or a waning of antibodies over time in the older age group in the absence of ongoing stimulation by infection. From longitudinal studies, it appears the antibodies are not reduced by treatment and are present for at least six months, but this was in the context of high rates of trachoma[11]. The rates of trachoma when the program started, as reported by the surveys done in Nepal, was 13.2% in Dang in 2003, and 11.6% in Dialekh in 2007, which were relatively low to begin with. It is possible that the low rate in the nine year olds in 2015 reflects very low to absent exposure growing up. Another study from 1997 in Nepal, in a neighboring district to Dialekh, found low trachoma rates of 6% but when testing 125 children for infection, none were positive[14].While this study does not have the power to detect infection levels at less than 1%, it suggests a long history of low trachoma in a neighboring district. We also recognize that there has been population movement into the Terai region of Nepal, which includes Dang, from north-eastern India where the rates of trachoma are unknown. As with any cross-sectional survey, the population evaluated were those currently in the area, but the antibody history may reflect exposure from outside Nepal.

Nevertheless, the absence of an increase by age in seroprevalence of antibodies to pgp3 antigens may also be a marker for interruption of transmission. In a study of a single sub-village in Tanzania where there was no infection, antibody positivity rate in the 200 children ages 1–9 years was 3·5%, compared to our finding of 2%[15]. In this sub-village, trachoma was 6·5%, whereas our trachoma rates were 3/1500 (0·2%). In a survey done as an impact assessment in Achham district in Nepal, low rates of infection and trachoma were also found, although too few children were tested for antibodies to determine a reliable rate of antibody positivity[16]. In the only other district surveillance prevalence survey, antibody prevalence in 30 communities in Kilosa district Tanzania was 7·5% and increased significantly with age from 5% in 1–3 year olds, to 9% in 7–9 year olds[9]. The higher rate in Kilosa may reflect the higher infection rate in this district, 1·1%, although the survey was done there 4 years after program cessation with no evidence of re- emergence of trachoma.

Another important component for surveillance is confirming a low rate of blinding TT in adults. Trichiasis due to trachoma will continue to manifest several years after active disease has been eliminated, and countries need to provide assurances that services for trachomatous trichiasis continue to be offered in formerly endemic districts. Ideally, as in Nepal, these services are integrated into existing programs. In Nepal, female community volunteers who carry out a variety of maternal and child health projects have been trained to screen for trichiasis and to refer cases to the district eye hospital. We found that it is important to confirm trachoma as the likely cause of trichiasis by looking for trachomatous scarring (TS) in the conjunctiva. In our survey we were able to characterize 4 of the 10 trichiasis cases in Dang district as unlikely due to trachoma and, while they were still offered services appropriate to their condition, they would not prevent the national program from reaching its elimination goal. While it may be that sub clinical scarring is associated with an aberrant lash[17], it is also likely that other causes of trichiasis could be the culprit and it seems unreasonable to attribute all trichiasis to trachoma and thus potentially prevent countries from reaching their elimination goals[18]. It is also unclear what the long term risk of vision loss is from non-scarring related trichiasis, since the overall goal is the elimination of blinding trachoma. We also found that the specific questions of being offered surgery before and refusing before, and again in this survey, helped in identifying trichiasis cases who are refusals and known to the health system. These cases again should not count against the trachoma trichiasis surgery elimination target, as patients do have a right to refuse services.

There are limitations to our study on antibody testing. Ideally, the determination of antibody status at the time of the impact survey immediately post-MDA would have enabled us to follow the trajectory of antibody responses over the time of surveillance. While we did not include the 5–8 year olds due to cost considerations, it is interesting that we found a similar seroprevalence rate in the 9 year olds as we did in the 1–4 year olds. A trajectory study would have enabled us to determine if this represents waning of antibodies in the older group, or a consistent, low level, exposure across all age groups. At present, the antibody test run on the Luminex platform is not readily available and technically complicated, but the designers are working on an ELISA and a field test version for pgp3 antibodies, which would bring it within the possibility of more widespread use.

Finally, the surveillance survey was powered to detect low prevalences of active disease at the district level, but the confidence intervals around the detection of TT at <1/1,000 are quite broad. In this study, the interval was 0·1 to 1·2%. There is also the concern that the TT cases may be more likely to be home on the day of the survey, and may preferentially be included in the random sample, inflating the estimate. With two thousand adults over age 14 and older in the district surveys, just 4 cases of TT might be sufficient to exceed the target of <1/1,000 total population, depending on the age distribution. Larger surveys of adults would address the precision issue, but come at a cost.

In summary, the new WHO surveillance surveys, carried out at 2 and 4 years post cessation of MDA in two districts in Nepal, showed no evidence for re-emergence of trachoma. The lack of significant age specific increase in seroprevalence of antibodies to *C*. *trachomatis*, and the low sero- prevalence, deserves further investigation as a marker of interruption of transmission. The survey also showed attainment of a goal of TT less than 1/1,000 population, but in one district with the addition of questions to determine previous surgical refusal, and flipping the lid to assess scarring and thus trachoma as the likely determinate of the trichiasis.

## Supporting Information

S1 Checklist STROBE checklist. (PDF)
